# Diverse potential of chimeric antigen receptor‐engineered cell therapy: Beyond cancer

**DOI:** 10.1002/ctm2.70306

**Published:** 2025-04-09

**Authors:** Lvying Wu, Lingfeng Zhu, Jin Chen

**Affiliations:** ^1^ Institute of Clinical Medicine The Second Affiliated Hospital of Hainan Medical University Haikou Hainan China; ^2^ Minimally Invasive Urology and Translational Medicine Center Fuzhou First General Hospital Affiliated With Fujian Medical University Fuzhou Fujian China

**Keywords:** autoimmune diseases, cardiac diseases, chimeric antigen receptor, infectious diseases, transplantation

## Abstract

**Background:**

Chimeric antigen receptor (CAR)‐engineered cell therapies have made significant progress in haematological cancer treatment. This success has motivated researchers to investigate its potential applications in non‐cancerous diseases, with substantial strides already made in this field.

**Main Body:**

This review summarises the latest research on CAR‐engineered cell therapies, with a particular focus on CAR‐T cell therapy for non‐cancerous diseases, including but not limited to infectious diseases, autoimmune diseases, cardiac diseases and immune‐mediated disorders in transplantation. Additionally, the review discusses the current obstacles that need to be addressed for broader clinical applications.

**Conclusion:**

With ongoing research and continuous improvements, CAR‐engineered cell therapy holds promise as a potent tool for treating various diseases in the future.

**Key points:**

- CAR‐engineered cell therapy has expanded beyond cancer to treat autoimmune diseases, infections, cardiac diseases, and transplant‐related rejection.
- The CAR platform is diverse, with various cell types such as CAR‐T, CAR‐NK, and CAR‐M potentially suited for different disease contexts.
- The safety, efficacy, and practicality of CAR cell therapy in non‐cancer diseases remain challenging, requiring further technological optimization and clinical translation.

## Graphical abstract

1. In theory, CAR‐engineering therapy can achieve therapeutic effects by targeting cells expressing specific antigens and thereby eliminating or regulating disease‐related cell subpopulations.

2. Although CAR‐engineering therapy is currently only approved for the treatment of cancer, it has also shown potential in non‐tumour diseases.

3. Immune cells from different sources expand the potential applications of CAR‐engineering therapy.

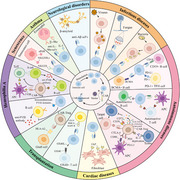

## 1 BACKGROUND

**FIGURE 1:**
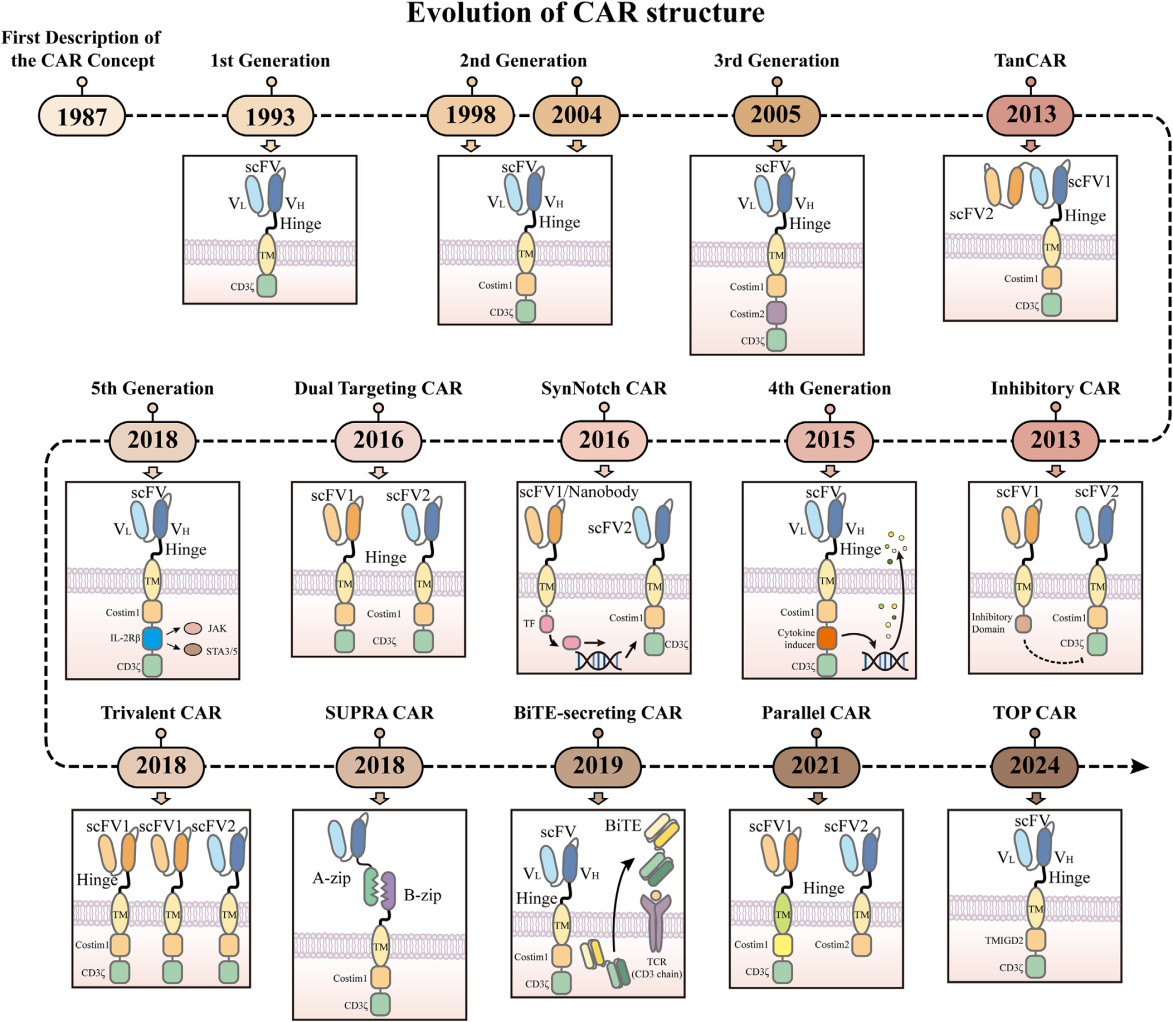
Illustration of the evolution of chimeric antigen receptor (CAR) structures. With continuous optimisation and improvements, the CAR structure has evolved to the fifth generation. Additionally, numerous CAR variants have been developed to enhance the specificity, safety, recognition capability and cytotoxicity of CAR‐engineered cells.

**FIGURE 2:**
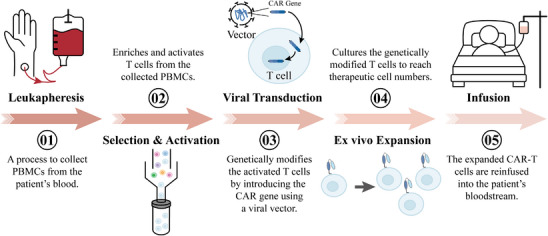
General process of chimeric antigen receptor (CAR) therapy. Peripheral blood mononuclear cell (PBMCs) are collected from the patient's peripheral blood, followed by the enrichment and activation of T cells. The CAR gene is then transduced into the activated T cells using viral vectors, and these genetically modified T cells are further expanded in vitro to reach therapeutic numbers. Finally, the genetically modified T cells in sufficient numbers are reinfused into the patient under appropriate conditions.

Chimeric antigen receptors (CARs) are receptor molecules manufactured through genetic engineering techniques that grant immune effector cells the ability to recognise specific antigens. Since the concept of CAR was first proposed in 1987,^1^ CAR structures have undergone multiple generations of development,^2^, ^3^, ^4^, ^5^, ^6^, ^7^, ^8^ with design advancements enhancing their specificity,^9^, ^10^, ^11^ safety^12^, ^13^, ^14^, ^15^ and functionality^16^, ^17^ (Figure 1). Efficient delivery of the CAR gene into immune effector cells is essential for achieving the functionality of different CAR designs, with major approaches including viral vector transduction and non‐viral delivery methods.^18^, ^19^ However, all seven CAR‐T cell products approved for clinical application, including Aucatzyl, a CD19‐targeted genetically modified autologous T cell therapy approved on 8 November 2024, for the treatment of relapsed or refractory B‐cell precursor acute lymphoblastic leukaemia, have utilised viral vector delivery.^20^, ^21^ Notably, although allogeneic and in vivo CAR‐T cells show significant promise, the CAR‐T therapies currently used in clinical practice are still autologous and manufactured ex vivo. This manufacturing process primarily encompasses several key stages, including T‐cell isolation, activation, viral transduction, CAR‐T cell expansion and reinfusion^22^, ^23^ (Figure 2).

As an effective immunotherapy, CAR‐engineered cell therapy has offered a glimmer of hope to cancer patients who have not benefited from most treatments. Among these, CAR‐T therapy has achieved remarkable success in addressing haematologic malignancies, with multiple products receiving regulatory approval.^24^, ^25^ Although some preclinical and clinical studies have preliminarily shown the potential of CAR‐T therapy for solid tumours,^15^, ^26^, ^27^ its application continues to face challenges such as tumour microenvironment suppression and antigen escape,^2^, ^28^ and no related products have yet been approved by major regulatory agencies. In contrast, CAR‐NK cells have attracted widespread interest for their inherent antitumour activity and immunomodulatory properties,^29^, ^30^ with their efficacy and safety in haematologic malignancies preliminarily validated in a clinical study (NCT03056339) conducted in 2020.^31^ Additionally, CAR‐M cell therapy, characterised by its superior tumour infiltration capabilities, tumour cell phagocytosis, antigen presentation and inflammatory cytokine secretion, is regarded as a novel strategy to overcome the immunosuppressive nature of solid tumours.^32^, ^33^ Although a preclinical study by Zhang et al. revealed that CAR‐M cells generated from induced pluripotent stem cells exhibited antitumour activity,^34^ their clinical efficacy and safety still require further validation.

The significant success of CAR‐engineered cell therapies for tumour treatment has prompted researchers to explore their potential for treating other diseases. In recent years, CAR‐engineered therapies have gained increasing attention for autoimmune diseases, transplant rejection, infectious diseases and cardiac diseases, with numerous clinical trials underway (Table 1). These diseases often involve complex pathological mechanisms, and existing treatment strategies face limitations in efficacy, safety or specificity. For example, in autoimmune diseases, traditional therapies such as hormones and immunosuppressants reduce the production of autoantibodies by systemically suppressing the immune system, but this broad immunosuppression often increases the risk of infections.^35^, ^36^ Conversely, CAR‐T cell therapies targeting autoreactive lymphocytes can specifically identify and eliminate pathogenic autoreactive B or T cells, thereby alleviating the disease without significantly compromising overall immune function.^37^ Additionally, regulatory T (Treg) cells are essential for immune suppression and the maintenance of immune homeostasis.^38^, ^39^ The development of antigen‐specific CAR‐Treg cells provides a targeted approach to suppressing immune responses implicated in autoimmune diseases.^39^, ^40^ Similarly, in transplant rejection, although the widespread use of immunosuppressants has significantly improved graft survival, their non‐specific immunosuppressive effects also increase the risk of infections and fail to completely prevent chronic rejection.^41^, ^42^ To address this challenge, CAR‐Treg cell therapies targeting graft‐specific antigens are considered a potential strategy for suppressing immune rejection, as they may achieve more precise and durable immune tolerance by modulating allogeneic immune responses.^43^, ^44^ In infectious diseases, while existing antiviral drugs can suppress viral replication, they often fail to completely eradicate viral reservoirs, and long‐term use may lead to drug resistance and side effects.^45^, ^46^ CAR‐T, CAR‐NK and CAR‐M cell therapies targeting pathogens exhibit potent antiviral activity and can eliminate latently infected cells.^47^, ^48^, ^49^ In the field of cardiac diseases, treatment options for cardiac fibrosis remain limited.^50^ CAR‐T and CAR‐M cell therapies designed to target antigens specific to activated cardiac fibroblasts offer a novel approach by specifically killing or phagocytosing fibroblasts, thereby mitigating cardiac fibrosis.^51^, ^52^ In brief, CAR‐engineered therapies, with their precise targeting and robust immunomodulatory capabilities, provide new possibilities for treating these diseases. This review aims to focus on the progress of CAR‐engineered cell therapy in fields beyond cancer.

**TABLE 1:** Summary of clinical trials using chimeric antigen receptor (CAR) therapy for non‐cancer diseases.

Category	Condition	Cell source	Target	Phase	Status	NCT
Infectious diseases	HIV infection	T cells	BNAbs	I	Unknown	NCT04863066
	HIV infection	T cells	gp120	I	Recruiting	NCT03240328
	HIV infection	T cells	gp120	Early I	Recruiting	NCT06252402
	HIV infection	T cells	gp120	I	Activate, not recruiting	NCT03617198
	HIV infection	T cells	gp120	I/II	Recruiting	NCT04648046
	COVID‐19	NK cells	S protein/ NKG2DL	I/II	Unknown	NCT04324996
Autoimmune diseases	ITP	T cells	BCMA	II	Unknown	NCT05315778
	SLE	T cells	CD19	I	Recruiting	NCT06150651
	SLE/SjS/SSc/DM/AAV	T cells	CD19	I	Recruiting	NCT06056921
	Scleroderma	T cells	CD19/BCMA	Early I	Recruiting	NCT05085444
	SjS	T cells	CD19/BCMA	Early I	Recruiting	NCT05085431
	Lupus nephritis	T cells	CD19/BCMA	Early I	Recruiting	NCT05085418
	SLE/SjS/SSc/IM/AAV	T cells	CD19	Not applicable	Recruiting	NCT06373081
	SLE	T cells	CD19/BCMA	Early I	Unknown	NCT05030779
	Crohn's disease/Ulcerative colitis/DM/Still diseases	T cells	CD7	Early I	Recruiting	NCT05239702
	Lupus nephritis	T cells	CD19	I/II	Recruiting	NCT06342960
	Lupus nephritis	T cells	CD19	I/II	Recruiting	NCT05938725
	MS	T cells	CD19	I	Recruiting	NCT06138132
	AAV/IIM/Lupus nephritis/SjS/SLE/SSc	T cells	CD19/BCMA	I/II	Not yet recruiting	NCT06350110
	MS	T cells	CD19	I	Recruiting	NCT06451159
	SLE/SjS	T cells	CD19/BCMA	I/II	Recruiting	NCT06428188
	AAV/IM/SjS/SLE/SSc/APS	T cells	CD19	Not applicable	Recruiting	NCT05859997
	SSc	T cells	CD19	I/II	Not yet recruiting	NCT06400303
	MG	T cells	CD19	I	Recruiting	NCT05828225
	MG	T cells	CD19	II	Not yet recruiting	NCT06193889
	Neuromyelitis optica	T cells	CD19	I	Recruiting	NCT05828212
	SLE/IMNM/NMOSD/MS	T cells	CD20/BCMA	I	Recruiting	NCT06249438
	DM	T cells	CD19	I	Not yet recruiting	NCT06298019
	SLE	T cells	CD19	Not applicable	Recruiting	NCT05988216
	AIHA	T cells	CD19	I	Recruiting	NCT06212154
	SLE	T cells	CD19	I	Unknown	NCT03030976
	MS	T cells	CD19	II	Not yet recruiting	NCT06384976
	SLE/Lupus nephritis	T cells	CD19	I	Not yet recruiting	NCT06544330
	IIM/SjS/SLE/SSc	T cells	CD19	Not applicable	Recruiting	NCT06361745
	IIM/MS/MG/NMOSD/MOGAD	T cells	BCMA	Early I	Recruiting	NCT04561557
	IIM/ITP/RA/SjS/SLE/SSc	T cells	CD19	Early I	Not yet recruiting	NCT06417398
	SLE	T cells	CD19	I	Recruiting	NCT06222853
	ITP	T cells	BCMA	Early I	Not yet recruiting	NCT06519565
	Lupus nephritis/SLE	T cells	CD19	I	Not yet recruiting	NCT06429800
	SLE	T cells	CD19/BCMA	I/II	Not yet recruiting	NCT06530849
	Pemphigus vulgaris	T cells	DSG3/CD19	I/II	Recruiting	NCT04422912
	Scleroderma/SSc	T cells	CD19	I/II	Recruiting	NCT06328777
	Lupus nephritis/SLE	T cells	CD19	I/II	Recruiting	NCT06121297
	SLE	T cells	CD19	I	Recruiting	NCT06333483
	MG	T cells	CD19	I/II	Recruiting	NCT06359041
	DM/IIM/IMNM	T cells	CD19	I/II	Recruiting	NCT06154252
	MuSk MG	T cells	MuSK	I	Recruiting	NCT05451212
	MG	T cells	BCMA	II	Recruiting	NCT04146051
	SLE	T cells	BCMA	II	Recruiting	NCT06038474
	SLE	T cells	CD19	I/II	Not yet recruiting	NCT06189157
	SLE	NK cells	CD19	I	Recruiting	NCT06518668
	IIM/SSc/SLE nephritis/AAV	T cells	CD19	I	Not yet recruiting	NCT06152172
	SSc/IIM/RA	NK cells	CD19	I	Recruiting	NCT06464679
	ITP	NK cells	CD19	Early I	Not yet recruiting	NCT06337474
	Immune nephropathy	NK cells	CD19	Early I	Recruiting	NCT06469190
	/	NK cells	CD19	Early I	Recruiting	NCT06318533
	SLE	NK cells	CD19	Early I	Recruiting	NCT06010472
	Lupus nephritis	NK cells	CD19	I	Not yet recruiting	NCT06377228
	SLE/IIM/SSc	T cells	CD19	I	Recruiting	NCT05869955
	MS	T cells	CD19	I	Recruiting	NCT06220201
Transplantation	Kidney transplantation	T cells	CD19/BCMA	I	Recruiting	NCT06056102
	Liver transplantation	Treg cells	HLA‐A2	I/II	Recruiting	NCT05234190
	Kidney transplantation	Treg cells	HLA‐A2	I/II	Active, not recruiting	NCT04817774
	Haematopoietic cell transplantation	Treg cells	CD6	I	Recruiting	NCT05993611

Abbreviations: AAV, anti‐neutrophil cytoplasmic antibody [ANCA]‐associated vasculitis; AIHA, autoimmune haemolytic anaemia; APS, antiphospholipid syndrome; DM, dermatomyositis; IIM, idiopathic inflammatory myopathy; IM, inflammatory myopathy; IMNM, immune‐mediated necrotising myopathy; ITP, immune thrombocytopenia; MG, myasthenia gravis; MOGAD, myelin oligodendrocyte glycoprotein antibody‐associated disease; MS, multiple sclerosis; NMOSD, neuromyelitis optica spectrum disorder; RA, rheumatoid arthritis; SjS, Sjogren's syndrome; SLE, systemic lupus erythematosus; SSc, systemic sclerosis. All data in this table are sourced from the ClinicalTrials database.

## 2 INFECTIOUS DISEASES

Although there have been significant advancements in controlling infectious diseases, infectious pathogens still pose a grave threat to the health of both humans and animals.^53^ T cells are crucial in both the pathogenesis and control of infectious diseases,^54^, ^55^, ^56^ prompting extensive preclinical and clinical research into the therapeutic applications of CAR‐T therapy in such conditions. Thus far, CAR‐engineered cell therapy has achieved a series of significant advancements in treating infectious diseases.

### 2.1 Viral infections

#### 2.1.1 Human immunodeficiency virus

**FIGURE 3:**
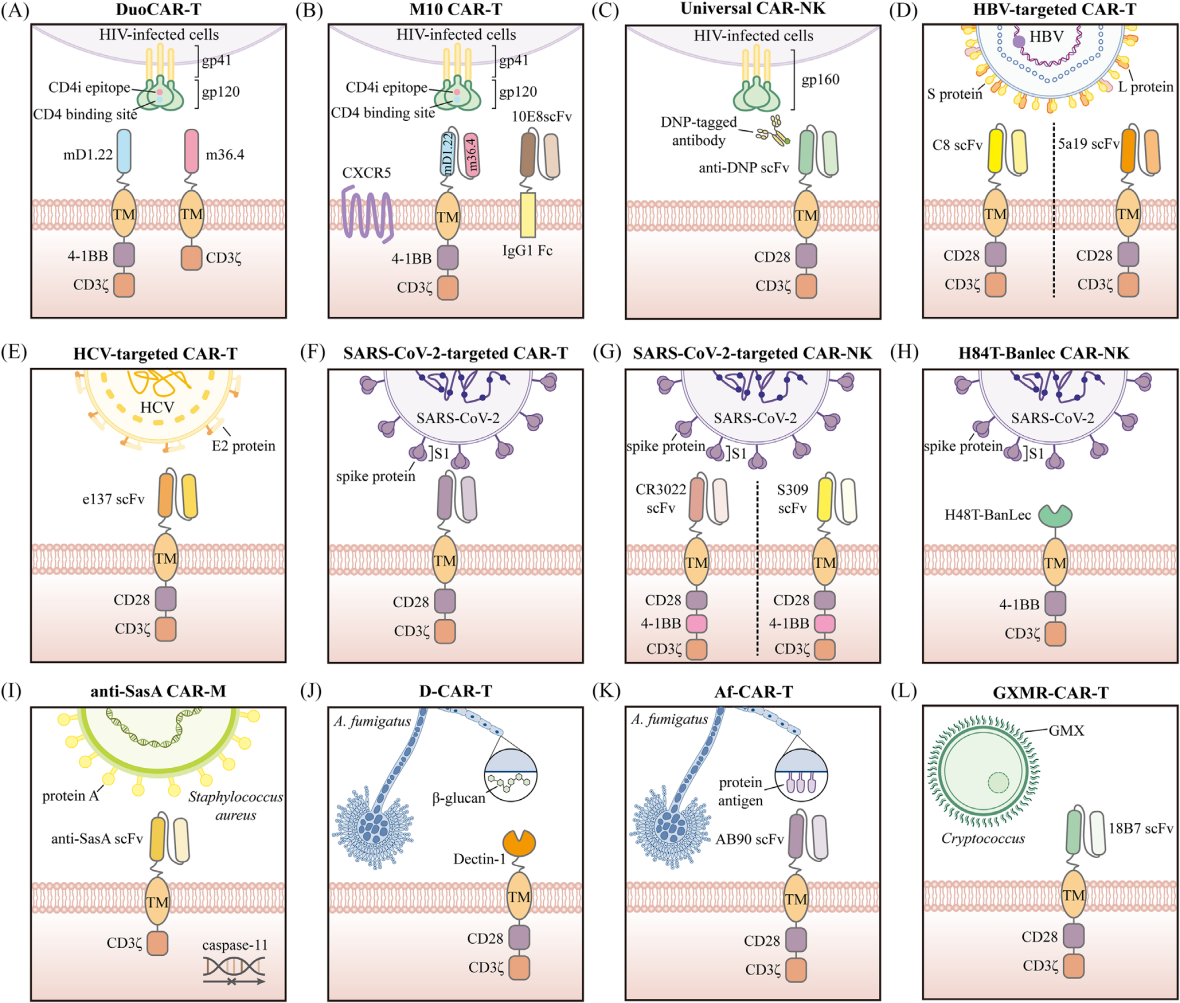
Schematic illustration of chimeric antigen receptor (CAR) constructs designed for infectious diseases. These constructs are designed to target various pathogen surface molecules, including proteins and polysaccharides, for the specific recognition and elimination of infected cells and pathogens.

Acquired immunodeficiency syndrome (AIDS), a chronic disease resulting from infection with the human immunodeficiency virus (HIV), has emerged as a significant global public health issue.^57^, ^58^ Despite antiretroviral therapy (ART) reducing the viral load in the plasma of HIV‐infected individuals to undetectable levels, ART alone is insufficient to completely eradicate the virus, as integration of HIV into the host cell genome leads to the formation of viral reservoirs.^59^, ^60^ Preclinical studies and early‐phase clinical trials have provided preliminary evidence supporting CAR‐T therapy in combating AIDS. Currently, there are two main strategies for designing CAR constructs targeting HIV infection: one that focuses on targeting the interaction between CD4 and gp120, and another that utilises broadly neutralising antibodies (BNAbs) to target the virus's envelope glycoproteins. The earliest CAR‐T cells designed for HIV infection involved the incorporation of the CD4 extracellular domain on CD8+ T cells, enabling them to bind to and lyse cells expressing HIV‐1 gp120 in vitro.^61^ However, despite the ability of these CAR‐T cells to inhibit viral replication, clear HIV‐infected cells, and persist for extended periods in vivo, they did not significantly impact the viral reservoirs.^62^ By incorporating the co‐stimulatory domain CD28, CD4‐CAR T cells effectively target and eliminate HIV Env‐expressing cells in cellular models and, through reactivation of latent HIV, identify and kill latently infected cells.^63^ Research based on an HIV‐infected humanised mouse model has shown that replacing the CD28 co‐stimulatory domain with the 4‐1BB co‐stimulatory domain not only greatly enhances CD4‐CAR T cells activity but also improves their ability to control HIV spread following ART treatment.^64^ CAR‐T therapies utilising BNAbs also exhibit effective antiviral activity,^65^ and ongoing improvements have enabled these cells to recognise and eliminate latently infected cells.^66^, ^67^ Additionally, bispecific CAR‐T cells combining CD4 and BNAbs have been investigated, showing stronger effects and more specific binding to target cells.^68^ Several clinical studies are assessing the safety and efficacy of CAR‐T therapy for treating AIDS.^69^, ^70^, ^71^ For example, Deeks et al. initiated a clinical trial (NCT04648046) aiming to recruit 18 individuals to evaluate the safety and anti‐HIV effects of T cells expressing bispecific anti‐gp120 CAR molecules (duoCAR; Figure 3A). In a prior preclinical study, they had already evaluated the capacity of duoCAR‐T cells to identify and destroy HIV‐infected cells in vitro and in vivo models, laying the groundwork for the current clinical trial.^69^ In June 2023, the research team provided a brief report on the first two patients treated with duoCAR‐T, with preliminary results indicating that the participants were in good condition and no related adverse events were observed.^70^ Additionally, another clinical trial led by Mao et al. aimed to assess the performance and safety profile of versatile M10 CAR‐T cells in treating HIV‐1‐infected patients.^71^ The multifunctional M10 CAR‐T cells are engineered to perform three key biological roles through the expression of a bispecific CAR molecule, a 10E8scFv‐Fc fusion antibody and the receptor CXCR5 for follicular homing. These functions include broad cytotoxicity against cells harbouring HIV, neutralisation of viruses in a cell‐free state and promotion of cell migration to germinal centres to target viral reservoirs (Figure 3B). The study demonstrated that, following M10 CAR‐T infusion, both plasma viral load and cell‐associated HIV‐1 RNA levels were significantly reduced, with no severe adverse events reported.

NK cells play a critical role in the innate immune response to viral infections.^72^ Compared to CAR‐T cells, CAR‐NK cells offer unique advantages, such as a lower risk of graft‐versus‐host disease (GVHD) and reduced long‐term toxicity.^73^ As a result, CAR‐NK cells are being explored as a viable option for HIV treatment. As early as the 1990s, researchers explored the use of retroviral transduction to genetically modify human NK cells for high expression of CD4ζ.^74^ This study demonstrated that these CD4ζ+ CAR‐NK cells could effectively lyse gp120‐expressing or HIV‐infected T cells. Unfortunately, in vivo experiments in mice revealed that CD4ζ+ CAR‐NK cells did not exhibit superior HIV‐suppressing efficacy compared to unmodified NK cells.^75^ Recently, Lim et al. designed a CAR‐NK cell capable of recognising multiple epitopes of gp160 from different HIV‐1 subtypes, potentially addressing the challenge of HIV diversity.^76^ This universal CAR‐NK cell expresses a CAR structure targeting 2,4‐dinitrophenyl (DNP) and utilises DNP‐conjugated anti‐gp160 antibodies as bridging molecules to target various gp160 epitopes (Figure 3C).

#### 2.1.2 Hepatitis virus

The hepatitis B virus (HBV) and hepatitis C virus (HCV) can lead to hepatitis, liver cirrhosis and even hepatocellular carcinoma.^77^ Antiviral treatments for chronic hepatitis B primarily rely on nucleos(t)ide analogues and interferons to inhibit the viral reverse transcriptase.^78^ Nucleos(t)ide analogues successfully inhibit HBV replication, yet the covalently closed circular DNA (cccDNA) of HBV remains in the nuclei of infected hepatocytes, posing a risk of viral relapse.^79^ Interferon therapy, though applicable in certain cases, is effective in only about 30% of patients and is associated with numerous side effects.^80^ The advent of direct‐acting antiviral agents (DAAs) has provided substantial hope in managing chronic hepatitis C.^81^ Nevertheless, HCV's genetic variability and resistance to DAAs remain challenges that need to be addressed. Recent studies have emphasised the advantages of CAR‐T therapy for combating HBV or HCV infections. Research by Bohne et al. showed that CAR‐T cells targeting HBV S or L proteins could recognise and eliminate HBsAg‐positive hepatocytes in vitro^82^ (Figure 3D). Further studies have confirmed the in vivo effectiveness and safety of CAR‐T therapy for HBV treatment in animal models.^83^, ^84^, ^85^ In addition, CAR‐T cells directed against the HCV E2 protein have shown the ability to lyse cells infected with various HCV subtypes, including 1a, 1b, 2a, 3a, 4 and 5, suggesting that CAR‐T therapy against HCV E2 could be an effective treatment for chronic HCV infection^86^ (Figure 3E).

#### 2.1.3 Human cytomegalovirus

In immunocompromised individuals, such as those with AIDS, transplant recipients and developing foetuses, human cytomegalovirus (HCMV) infection is linked to elevated morbidity and mortality rates.^87^, ^88^ Adoptive transfer therapy using ex vivo expanded autologous cytomegalovirus (CMV)‐specific cytotoxic T lymphocytes (CTLs) represents a feasible approach^89^, ^90^, ^91^; however, it is limited by the complexity of antigen‐specific CTL expansion techniques in vitro. Additionally, this method relies on human leukocyte antigen (HLA) matching, which restricts its broad applicability across diverse patient populations. CAR‐engineered therapies hold promise for overcoming these limitations. The earliest CAR constructs designed for CMV infection targeted the CMV glycoprotein B (gB) and incorporated the CD28 co‐stimulatory domain.^92^ These gB‐CAR‐T cells, when tested in vitro and stimulated by CMV‐infected human foreskin fibroblast (HFF) cells, produced interferon‐gamma (IFN‐γ) and tumour necrosis factor (TNF) while effectively eliminating gB‐positive target cells at low effector‐to‐target ratios. Subsequently, Olbrich et al. compared the impact of different co‐stimulatory domains on the antiviral activity of gB‐CAR‐T cells.^93^ Their findings indicated that CAR‐T cells containing the 4‐1BB co‐stimulatory domain demonstrated stronger antiviral activity in vitro than those with the CD28 co‐stimulatory domain. Furthermore, they validated the in vivo efficacy of gB‐CAR‐T cells with the 4‐1BB co‐stimulatory domain using a humanised mouse model, demonstrating that five out of eight mice showed a significant therapeutic response. Recently, Ali et al. designed a series of novel CARs targeting different CMV proteins and identified that the 21E9 monoclonal antibody (mAb)‐based CAR‐T cells targeting gH exhibited the strongest anti‐CMV activity in vitro.^94^ However, the in vivo efficacy and safety of those CAR‐T cells still require further validation.

#### 2.1.4 Epstein–Barr virus

Epstein–Barr virus (EBV), a member of the human B‐lymphotropic herpesvirus family, is highly prevalent, with an infection rate exceeding 95%.^95^ Typically, EBV can establish lifelong latency without causing severe clinical symptoms; however, in immunocompromised individuals, EBV may reactivate and lead to various life‐threatening diseases.^96^ A previous clinical study demonstrated that adoptive transfer of EBV‐specific CTLs is effective in managing EBV‐positive lymphoproliferative disease (EBV‐LPD) after bone marrow transplantation,^97^ while another clinical study showed that this approach exhibits antitumour effects in some patients with EBV‐associated malignancies.^98^ These findings suggest that CAR‐T cells designed to recognise EBV‐associated antigens may also hold therapeutic potential. Researchers have found that CAR‐T cells designed against EBV latent membrane protein 1 (LMP1) and envelope glycoprotein 350 (gp350) effectively inhibit tumour growth in mouse models.^99^, ^100^ Additionally, IL‐15/IL‐15Rα‐NKG2D CAR‐T cells have shown potent antiviral and antitumour activity against EBV‐associated post‐transplant lymphoproliferative disorder (EBV‐PTLD) by promoting central memory T cell expansion, improving in vivo homing and persistence and increasing the secretion of IFN‐γ, perforin and granzyme.^101^ While these preclinical results are encouraging, further research is needed to clarify the clinical effectiveness of CAR‐T cells in EBV‐associate conditions.

#### 2.1.5 Severe acute respiratory syndrome coronavirus 2

Another emerging field of research is focusing on deploying CAR‐T therapy for severe acute respiratory syndrome coronavirus 2 (SARS‐CoV‐2) infections. Guo et al. conferred upon CAR‐T cells the ability to recognise the receptor‐binding domain peptide of SARS‐CoV‐2 (Figure 3F), enabling them to exhibit strong cytotoxic effects against S1‐expressing cells.^102^ As mentioned earlier, the critical role of NK cells in viral infections, combined with the unique advantages of CAR‐NK therapy, has spurred extensive research into the application of CAR‐NK therapy for treating SARS‐CoV‐2 infection. Two research groups independently utilised the scFv domains of neutralising antibodies (CR3022 and S309) to construct CAR‐NK cells targeting SARS‐CoV‐2^103^, ^104^ (Figure 3G). These CAR‐NK cells equipped with scFv domains from neutralising antibodies exhibited the ability to target and kill SARS‐CoV‐2‐infected cells in vitro. Subsequently, the antiviral efficacy of S309‐based CAR‐NK cells was further validated in vivo.^105^ These S309‐CAR‐NK cells significantly reduced the SARS‐CoV‐2 viral load in the lungs of NOD/SCID γ mice expressing the human angiotensin‐converting enzyme 2 (hACE2) receptor. In addition to neutralising antibodies, Christodoulou et al. designed CAR‐NK cells that extracellularly express H84T‐banana lectin (H84T‐Banlec).^106^ Previous studies demonstrated that H84T‐Banlec could bind to high‐mannose glycans, enabling its remarkable antiviral activity with potential therapeutic applications against multiple pathogenic coronaviruses.^107^ Consistent with these findings, H84T‐Banlec CAR‐NK cells (Figure 3H) were able to reduce the infection of S‐protein pseudotyped lentivirus in ACE2‐expressing 297T cells in vitro. Lu et al. took a different approach by engineering NK cells to directly express the extracellular domain of the SARS‐CoV‐2 target protein ACE2 and secrete IL‐15.^108^ These engineered CAR‐NK cells could be specifically activated by SARS‐CoV‐2 and produce TNF‐α and IFN‐γ, ultimately enhancing their cytotoxicity against S protein‐expressing cells in both in vitro and in vivo. As key effector cells of the innate immune system, macrophages possess the dual functionality of sensing and responding to microbial threats while promoting tissue repair, which led Fu et al. to hypothesise that CAR‐M cells could be utilised to combat SARS‐CoV‐2.^49^ Similar to CAR‐NK therapies based on neutralising antibodies, they engineered human macrophages using scFv derived from CR3022 to confer phagocytic activity against SARS‐CoV‐2.^49^ However, instead of conventional CAR designs, they utilised the MER proto‐oncogene tyrosine kinase (MERTK) as the intracellular signalling domain of the CAR. This design enabled CAR_MERTK_‐Ms to exhibit antigen‐dependent phagocytosis of viral particles while avoiding the induction of inflammation.

In summary, CAR‐engineered therapies are increasingly being explored for viral infections, highlighting their versatility and potential to revolutionise the treatment of various viral diseases. Optimising CAR‐engineered cell design, improving efficacy and ensuring safety necessitate further research and clinical trials.

### 2.2 Bacterial infections

Bacterial infections have long been one of the most serious challenges in medicine, with antibiotics serving as an effective means of treatment.^109^ However, the rising prevalence of antibiotic‐resistant bacteria and the limitations of current antibiotic therapies underscore the urgent need for innovative approaches to treating bacterial infections.^110^ Currently, research on CAR‐T therapy for bacterial infections is quite limited, with its most promising application being the treatment of chronic diseases such as tuberculosis. Although CAR cells have not yet been developed for tuberculosis, several potential strategies have been proposed. One approach involves identifying antibodies against bacterial antigens in latently infected tuberculosis patients, which could then be cloned to generate scFv fragments and ultimately used to create CARs for T cells and NK cells.^111^ Additionally, given the critical role that NKT cells play in tuberculosis infection,^112^, ^113^, ^114^ researchers have suggested their direct use in adoptive cell therapy or the incorporation of their receptors as recognition domains in CAR constructs.^115^ Another promising avenue is the development of CAR‐NKT cells, which could utilise both natural receptors and CARs to recognise bacteria, thereby overcoming potential mutations in antigens recognised by the CAR.^115^ Notably, Liang et al. reported the safety and therapeutic effectiveness of Vγ9Vδ2 T cells in treating tuberculosis in a clinical study.^116^ This finding indicates that Vγ9Vδ2 T cells may be further enhanced through integration with CAR technology, offering improved efficacy in complex infectious environments. Encouragingly, while evidence supporting CAR‐T therapy for bacterial infections remains limited, the efficacy of CAR‐M therapy has been preliminarily validated. Given the critical role of macrophages in host defence and inflammation regulation during microbial infections,^117^, ^118^, ^119^ as well as their importance in the innate immune response to *Staphylococcus aureus* (*S. aureus*),^120^, ^121^ researchers have attempted to engineer CAR‐M cells targeting *S. aureus*‐specific antigens to enhance their phagocytic and bactericidal capabilities against this pathogen. For instance, Li et al. developed a nanoparticle coating that enables in vivo delivery of a CAR targeting *S. aureus* surface protein A (SasA) and caspase‐11 shRNA, ultimately leading to the localised induction of CAR‐M cells in the prosthetic microenvironment^122^ (Figure 3I). These anti‐SasA CAR‐M cells exhibited dual functionalities: targeting and phagocytosing *S. aureus* through the expression of anti‐SasA scFv, while suppressing caspase‐11 expression, which allowed mitochondrial recruitment around phagosomes to produce reactive oxygen species for bacterial killing. Similarly, Tang et al. designed lipid nanoparticles modified with sequence CRVLRSGSC (CRV) peptides to encapsulate and deliver SasA‐CAR mRNA and caspase‐11 siRNA into macrophages, effectively generating CAR‐M cells with potent bactericidal activity.^123^

### 2.3 Fungal infections

Fungal infections annually cause around 11.5 million life‐threatening cases and over 1.5 million deaths,^124^ posing a significant threat to public health. At present, antifungal drugs targeting fungal pathogens, including azoles, echinocandins, polyenes, allylamines and antimetabolites, remain the first‐line treatment for infected patients.^125^ However, due to the continuous emergence of resistance, these drugs are becoming increasingly ineffective at treating the growing number of fungal infections, with the mortality rate for invasive fungal infections remaining high.^126^ The prospects of CAR‐T therapy in treating fungal infections have been explored in preliminary studies. Kumaresan et al. engineered CAR‐T cells to incorporate the pattern recognition receptor Dectin‐1, which can trigger T cell activation through chimeric CD28 and CD3‐ζ upon engagement with carbohydrates present in the cell wall of *Aspergillus fumigatus* (Af) germlings (Figure 3J), ultimately inhibiting the growth of Af both in vitro and in vivo.^127^ Another study designed and constructed Af‐specific chimeric antigen receptor (Af‐CAR) T cells targeting a conserved protein antigen in the cell wall of Af (Figure 3K), demonstrating their antifungal activity in preclinical models. These Af‐CAR‐expressing T cells can recognise Af strains and directly target Af hyphae with antifungal effects.^128^ Additionally, CAR‐T cells engineered to target glucuronoxylomannan (GXM) (Figure 3L) have shown their potential to recognise both *Cryptococcus neoformans* and *Cryptococcus gattii*.^129^

## 3 AUTOIMMUNE DISEASES

Autoimmune diseases rank as the third most prevalent type of disease, following cancer and cardiovascular disease, affecting nearly 10% of the global population.^130^ Currently, approximately 100 autoimmune diseases have been identified.^131^ Despite the significant differences in the clinical manifestations of these autoimmune diseases, they share a common feature: an imbalance in immune tolerance that gives rise to the formation of autoreactive T cells, autoreactive B cells and autoantibodies against self‐antigens.^132^ Steroids, potent anti‐inflammatory drugs, cytotoxic drugs, anti‐rheumatic drugs and monoclonal antibodies targeting specific cells or autoantibodies are the main therapeutic approaches currently used for autoimmune diseases. However, these treatments are associated with significant side effects and can only alleviate symptoms without achieving a cure.^133^ In light of the outstanding achievements of CAR‐T therapy in haematologic cancers, researchers are now exploring its potential applications in the field of autoimmune diseases. It is noteworthy that recently published guidelines on the application of innovative cellular therapies, including CAR‐T cells, in autoimmune diseases provide specific recommendations regarding indications, treatment strategies and safety management, offering valuable references for clinical practice.^134^

### 3.1 Systemic lupus erythematosus

**FIGURE 4:**
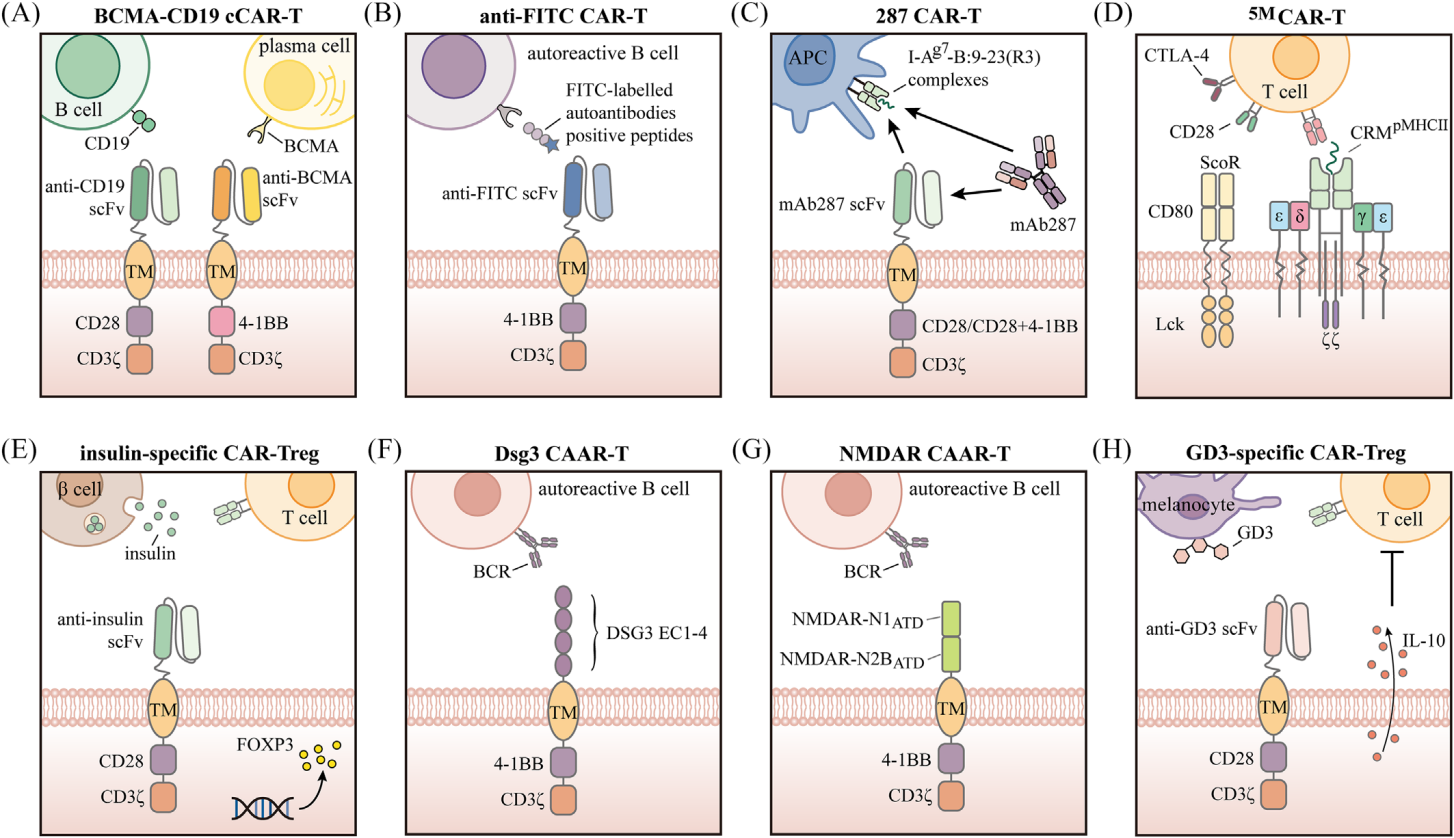
Schematic illustration of chimeric antigen receptor (CAR) constructs designed for autoimmune diseases. These CAR constructs are expressed on the surface of T cells or Treg cells, enabling them to specifically target, eliminate or suppress B cells, antigen‐presenting cells, T cells and other immune cells involved in the autoimmune response.

A hallmark of systemic lupus erythematosus (SLE) is the generation of antinuclear antibodies (ANAs), which form immune complexes, activate complement and promote the release of inflammatory cytokines, leading the immune system to mistakenly target the body's healthy tissues and cells.^135^ B cells play a crucial role in the development of SLE due to their secretion of autoantibodies and pro‐inflammatory cytokines, making them a key therapeutic target in the disease.^136^ CD19 is a key regulator of B‐cell differentiation, antibody production and memory formation, and is expressed on all mature B cells, memory B cells and plasmablasts, as well as on certain pre‐B cells and plasma cells.^137^ Utilising CD19 for deep B‐cell depletion could reset the immune system, potentially leading to long‐lasting therapeutic effects in autoimmune diseases, including SLE.^132^ Previous studies have offered compelling evidence supporting that CD19‐targeted CAR‐T cell therapies are both effective and safe in SLE‐related animal models.^138^, ^139^ Even more excitingly, the first case of an SLE patient being treated with CD19 CAR‐T cells was documented in 2021.^140^ Post‐treatment, the patient's autoantibodies disappeared, their condition improved, and no significant side effects were observed. Subsequently, the same research team reported results in 2022 for an additional five refractory SLE patients receiving treatment with CD19 CAR‐T cells.^141^ The results showed that all patients reached remission of SLE 3 months after treatment, and during follow‐up periods of up to 17 months, drug‐free remission was maintained even after B cells reappeared. Most recently, this group published a follow‐up case series.^142^ This study tracked 15 patients treated with CD19 CAR‐T cells between February 2021 and May 2023, including those with severe SLE, systemic sclerosis (SSc) and idiopathic inflammatory myositis, some of whom had been previously reported. The results indicated that all patients experienced disease improvement and tolerated the treatment well, suggesting that CAR‐T therapy represents a viable option for these three distinct autoimmune diseases. The significantly increased expression of B‐cell maturation antigen (BCMA) across all B‐cell subsets in SLE patients makes it a candidate therapeutic target.^143^ In another small phase I study, the CD19/BCMA dual‐target CAR‐T cell therapy (Figure 4A) developed by iCell Gene Therapeutics safely induced drug‐free remission in SLE patients.^144^ Of particular note is that the first paediatric SLE patient undergoing CD19‐targeted CAR‐T therapy was described in 2024.^145^ This patient, who had early‐onset and rapidly progressive lupus nephritis, experienced a rapid decline in SLE disease activity, resolution of arthritis symptoms and improvement in renal function following CAR‐T therapy. These findings suggest that paediatric SLE patients may also benefit from CAR‐T therapy, highlighting the urgent need for further clinical trials.

The deficiency of Treg cells may play a critical role in the pathogenesis of SLE,^146^ while antigen‐specific Treg cells have shown greater efficacy and safety than polyclonal Tregs in preclinical studies.^147^ These findings collectively provide a theoretical foundation for CAR‐Treg cell therapy in SLE. Building on this foundation, Doglio et al. developed Treg cells co‐expressing an anti‐CD19 CAR and FoxP3 (Fox19CAR‐Tregs) for the treatment of SLE.^148^ In vitro experiments demonstrated that these Fox19CAR‐Tregs effectively suppressed B‐cell proliferation and activation, while in a humanised mouse model of SLE, they reduced autoantibody production and promoted the restoration of immune homeostasis.

Although CAR‐T therapy has demonstrated significant efficacy in autoimmune diseases and severe toxicities are rarely reported in treated patients, the limited number of autoimmune patients treated to date necessitates cautious interpretation of the available safety data.^149^ In contrast, CAR‐NK cells exhibit clear safety advantages, with early clinical trials reporting minimal severe side effects like cytokine release syndrome (CRS) or neurotoxicity.^31^, ^150^ Additionally, sources such as umbilical cord blood, stem cells and cell lines can be used to generate CAR‐NK cells, not only reducing manufacturing costs but also enhancing clinical feasibility.^151^, ^152^ Leveraging these advantages, researchers have started investigating CAR‐NK therapy as a treatment option for SLE. A research team in the United States engineered NK‐92 cells, a human NK cell line, to express a CAR construct containing the extracellular domain of PD‐L1.^153^ These modified CAR‐NK cells were designed to target and eliminate PD‐1‐expressing follicular helper T (TFH) cells, which have been implicated in the pathogenesis of various autoimmune diseases.^154^ In both in vitro studies and lupus‐like humanised mouse models, PD‐L1‐CAR‐NK cells selectively eliminated PD‐1‐high CD4+ T cells, including TFH cells, leading to a reduction in memory B‐cell proliferation, differentiation and Ig antibody secretion.

### 3.2 Rheumatoid arthritis

Rheumatoid arthritis (RA) is a chronic, systemic autoimmune disease that mainly impacts joints and soft tissues surrounding them.^155^ B cells are pivotal in the development of RA, and rituximab, a monoclonal antibody that targets CD20 antigen and results in the depletion of B cells, can significantly improve the symptoms and signs in RA patients. However, rituximab is linked to an elevated risk of infection and requires long‐term treatment cycles.^156^ Zhang et al. found that CAR‐T cells engineered to target FITC (Figure 4B) can selectively eliminate hybridoma cells derived from antigen peptide immunisation, as well as autoreactive B cells from RA patients, through the recognition of corresponding FITC‐tagged citrullinated peptide epitopes.^157^ Recently, a clinical trial employing CD19‐targeted CAR‐T cells for RA treatment demonstrated that, despite early adverse events, the patient achieved drug‐free remission by day 100 without any neurological sequelae.^158^

### 3.3 Systemic sclerosis

SSc is an uncommon and complex autoimmune disorder marked by progressive connective tissue fibrosis, microvascular damage, innate and adaptive immune dysregulation, and multi‐organ or systemic fibrosis.^159^ At present, there is no cure for SSc, and treatment primarily aims to alleviate symptoms, reduce functional disability and improve quality of life.^160^ The imbalance of B cells in SSc patients, along with the observed improvements in lung function and skin fibrosis with rituximab treatment, suggests that targeting CD19 with CAR‐T cells might be a useful strategy.^161^ In May 2023, Georg Schett's team administered CD19 CAR‐T therapy to an individual diagnosed with severe, refractory SSc involving the skin, joints, heart and lungs. Following treatment, improvements were observed in the patient's skin hardening, cardiac and joint damage and serological markers.^162^ Subsequently, the results of two clinical studies further supported the therapeutic potential and safety of CD19‐targeted CAR‐T therapy in SSc.^163^, ^164^ Both studies demonstrated that patients with SSc treated with CD19‐targeted CAR‐T cells achieved disease remission, with no severe adverse events observed.

### 3.4 Type 1 diabetes

Type 1 diabetes (T1D) is a chronic autoimmune disease where pancreatic β‐cells are destroyed, causing a shortage of insulin.^165^ Initial studies have suggested the potential practicality of CAR‐based therapies for treating T1D. In previous research, Zhang et al. produced a monoclonal antibody named mAb287, which selectively recognises complexes formed by the major autoantigen insulin peptide (B:9‐23) and major histocompatibility complex (MHC) class II molecules (I‐A^g7^) in register 3 (R3) in non‐obese diabetic (NOD) mice.^166^ Through this mechanism, mAb287 blocks the binding of I‐A^g7^‐B:9‐23(R3) complexes to T cells, thereby inhibiting the activation of insulin‐specific CD4 T cells and significantly delaying the onset of diabetes, without affecting the presentation of other antigens. Subsequently, the team further introduced this antibody into a CAR structure, creating cytotoxic 287‐CAR‐T cells (Figure 4C). These cells can selectively bind to specific I‐A^g7^‐B:9‐23(R3) complexes and eliminate the corresponding antigen‐presenting cells, thereby delaying the development of T1D.^167^ Additionally, Kobayashi et al. described a first‐generation biomimetic five‐module chimeric antigen receptor (^5M^CAR) aimed at targeting autoimmune CD4+ T cells in a diabetic mouse model. This biomimetic ^5M^CAR comprises a chimeric receptor module, three cluster of differentiation (CD) signalling modules and a surrogate co‐receptor module (Figure 4D). The chimeric receptor module is constructed by fusing the antigen targeted by pathogenic T cells with T‐cell receptor (TCR)‐associated elements, while the surrogate co‐receptor module is formed by the fusion of CD80 and Lck to enhance signalling transduction. The results showed that ^5M^CAR‐T cells were capable of both preventing and alleviating T1D in NOD mice and persisting in vivo for an extended period.^168^

The functional impairment of Tregs in patients with T1D suggests their potential involvement in the pathogenesis of the disease.^169^, ^170^ Adoptive transfer of Tregs is considered a potential therapeutic strategy^171^, ^172^, ^173^; however, studies have shown that islet antigen‐specific Tregs are significantly more effective than polyclonal Tregs in preventing or delaying disease progression.^174^, ^175^, ^176^, ^177^ Nevertheless, due to the limited availability of antigen‐specific Tregs under autoimmune conditions, their isolation and expansion pose significant challenges. CAR engineering offers an ideal solution to overcome this limitation. Tenspolde et al. proposed the first CAR‐Treg designed to improve and treat T1D by transducing a second‐generation CAR and the FOXP3 gene into CD4+ T cells, resulting in insulin‐specific CAR‐Treg cells^178^ (Figure 4E). These insulin‐specific CAR‐Treg cells exhibited a phenotype similar to that of natural Treg cells and effectively suppressed antigen‐specific T cells in vitro. Unfortunately, their infusion into NOD mice did not prevent the onset of diabetes. This lack of efficacy may be related to the secretion form and metabolic rate of insulin in vivo, suggesting that selecting more appropriate targets may be necessary. In another study, Spanier et al. developed CAR‐Treg cells targeting the insulin B chain 10–23 peptide presented by the I‐Ag7 MHC II allele and demonstrated that these cells effectively prevented disease onset in a mouse model of diabetes.^179^

### 3.5 Immune thrombocytopenia

Immune thrombocytopenia (ITP) is an autoimmune bleeding disorder characterised by a reduced platelet count in peripheral blood, mainly caused by enhanced platelet clearance and reduced platelet production.^180^ Among the underlying mechanisms, B‐cell‐derived autoantibody‐mediated platelet destruction is considered a key driver of ITP pathogenesis.^181^ In July 2024, the first case of SLE‐associated ITP managed using CD19‐targeted CAR‐T cell therapy was reported.^182^ The patient, diagnosed with SLE, presented with persistent thrombocytopenia. After receiving CAR‐T cell therapy, the patient showed a substantial rise in platelet count alongside a notable decrease in ANA titres. This case suggests that CD19‐targeted CAR‐T therapy may be effective in treating SLE‐associated ITP and further implies its potential application in primary ITP. This hypothesis has recently received preliminary validation. A German research team reported a case of primary ITP treated with CD19‐targeted CAR‐T cells.^183^ After treatment, the patient's platelet count increased significantly, and no recurrence of ITP was observed for approximately 3 months after discontinuation of all therapies, with platelet counts remaining within the normal range.

### 3.6 Inflammatory bowel disease

Inflammatory bowel disease (IBD) represents a category of progressive, chronic inflammatory conditions affecting the gastrointestinal tract, classified as autoimmune disorders, with the main types being Crohn's disease and ulcerative colitis.^184^, ^185^ Over the past decades, the incidence of IBD has risen sharply, with a trend towards an earlier age of onset.^186^ Unlike most autoimmune diseases, the therapeutic potential of CD19‐targeted CAR‐T therapy in IBD remains unclear, which may be related to the fact that the positive role of CD20 antibodies in IBD has not yet been proven.^187^ However, given that Treg cell dysfunction is considered a key mechanism in IBD pathogenesis,^188^, ^189^ researchers have actively explored the potential of CAR‐Tregs in IBD and have made preliminary progress. As early as 2008, Elinav et al. demonstrated that CAR‐Tregs specifically targeting 2,4,6‐trinitrophenol (TNP) could be activated by exogenous TNP in a 2,4,6‐trinitrobenzenesulfonic acid (TNBS)‐induced colitis mouse model, accumulate at colitis lesion sites and ameliorate intestinal inflammation by suppressing effector T cells.^190^ Given the elevated expression of carcinoembryonic antigen (CEA) in human colitis and colorectal cancer, Blat et al. designed CAR‐Tregs targeting CEA and validated their ability to reduce colitis severity and inhibit colorectal cancer development in mouse models.^191^ Furthermore, Cui et al. developed CAR‐Tregs targeting the interleukin‐23 receptor (IL23R).^192^ Their findings showed that these IL23R‐CAR‐Tregs could migrate to IL23R‐expressing tissues in humanised mice and be activated in vitro by cells derived from intestinal biopsies of Crohn's disease patients.

### 3.7 Neuromyelitis optica spectrum disorder

Neuromyelitis optica spectrum disorder (NMOSD) is an uncommon autoimmune disease of the central nervous system that often results in severe sequelae or even death.^193^ Its characteristic features include recurrent acute episodes and the detection of antibodies specific to the AQP4 protein, namely, AQP4‐IgG.^193^ B cells contribute to the pathogenesis of NMOSD through mechanisms such as maturing into plasmablasts and plasma cells that produce pathogenic AQP4‐IgG, secreting pro‐inflammatory cytokines like IL‐6 that enhance inflammatory immune responses, and functioning as antigen‐presenting cells to activate autoimmune T cells.^194^ Given the excellent performance of CAR‐T therapy in eliminating B cells, it is being considered for the treatment of NMOSD. Qin et al. reported a preliminary clinical study using CAR‐T cells targeting BCMA in a cohort of 12 patients with recurrent or refractory AQP4‐IgG seropositive NMOSD. The results indicated that CAR‐T cell therapy was well tolerated and exhibited a favourable safety profile, with 11 of 12 patients remaining relapse‐free over a median follow‐up period of 5.5 months. Considerable improvements in functional scores and quality of life were also observed.^195^

### 3.8 Myasthenia gravis

Myasthenia gravis (MG) is an autoimmune disease that impairs neuromuscular junction (NMJ) transmission, primarily driven by antibodies targeting acetylcholine receptors (AChR), muscle‐specific kinase (MuSK) or other AChR‐associated proteins expressed on the postsynaptic muscle membrane.^196^ Recently, Granit's research team utilised RNA transfection to develop CAR proteins targeting BCMA, which can be continuously expressed over the course of a week for the treatment of MG, aiming to reduce the inherent risks of conventional CAR‐T cell therapy. The study concluded that this therapy significantly improved the disease condition in MG patients without causing serious adverse reactions.^197^ The 12‐month follow‐up results of patients receiving this treatment were further reported in a preprint.^198^ The data showed that five out of seven participants maintained clinical improvement at 12 months.

### 3.9 Pemphigus vulgaris

Pemphigus is a chronic autoimmune blistering disease that poses a life‐threatening risk, primarily due to autoantibodies targeting desmoglein (DSG) 1 and DSG3.^199^ Pemphigus vulgaris (PV) is the predominant type of pemphigus, representing more than 80% of cases.^200^ A previous study demonstrated that DSG3 chimeric autoantibody receptor T (DSG3‐CAART) cells (Figure 4F), which are engineered T cells expressing a chimeric autoantibody receptor composed of the PV autoantigen DSG3 and CD137‐CD3ζ signalling domains, specifically targeted B lymphocytes expressing DSG3 B‐cell receptors (BCRs) in vitro and showed expansion and persistence in vivo.^201^ Further research by Lee et al. found that DSG3‐CAART cells could specifically lyse anti‐DSG3 human B cells from PV patients. In an active immune model of PV with physiological levels of anti‐DSG3 IgG, these DSG3‐CAART cells effectively suppressed antibody responses targeting pathogenic DSG3 epitopes and blocked the binding of autoantibodies to epithelial tissues, ultimately achieving clinical and histological resolution of blisters.^202^

### 3.10 Other autoimmune diseases

In antisynthetase syndrome (ASS), Georg Schett's team reported on a patient who underwent CD19 CAR‐T therapy after failing to respond to multiple immunosuppressants and biologics. After treatment, the patient's condition significantly improved, with nearly complete resolution of pulmonary, joint and muscle inflammation, and gradual recovery of muscle strength and endurance.^203^ Subsequently, Pecher et al. reported another successful case of ASS managed with CD19 CAR‐T therapy.^204^ In autoimmune encephalitis, Reincke et al. developed N‐methyl‐D‐aspartate receptor (NMDAR)‐CAART cells (Figure 4G) to selectively eliminate anti‐NMDAR B cells and pathogenic autoantibodies.^205^ In juvenile dermatomyositis, a case report published by Nicolai et al. demonstrated that the patient achieved significant clinical remission starting at week 4 after receiving a single dose of CD19‐targeted CAR‐T cells, and this remission persisted even after B‐cell recovery.^206^ In addition, recent advancements have been made in applying CAR‐T therapy for multiple sclerosis (MS) treatment. Kyverna Therapeutics first reported the use of KYV‐101, a fully human CD19 CAR‐T therapy, in two patients with progressive MS.^207^ Following treatment, one patient showed a significant reduction in intrathecal antibody production in the cerebrospinal fluid, and neither patient exhibited severe adverse reactions. To further collect data, Kyverna Therapeutics is conducting clinical trials independently or in collaboration (NCT06138132, NCT06384976). CAR‐Tregs are now being investigated for their potential in treating autoimmune skin disorders, with vitiligo being a notable example. Mukhatayev et al. explored the use of GD3‐specific Treg cells (Figure 4H) for the treatment of vitiligo,^208^ based on previous studies demonstrating that ganglioside D3 (GD3) is upregulated in perilesional epidermal cells, including melanocytes.^209^ They found that GD3‐specific Treg cells exhibited enhanced IL‐10 secretion upon antigen stimulation, effectively controlled cytotoxic responses against melanocytes and significantly delayed depigmentation in a spontaneous vitiligo mouse model.

## 4 CARDIAC DISEASES

**FIGURE 5:**
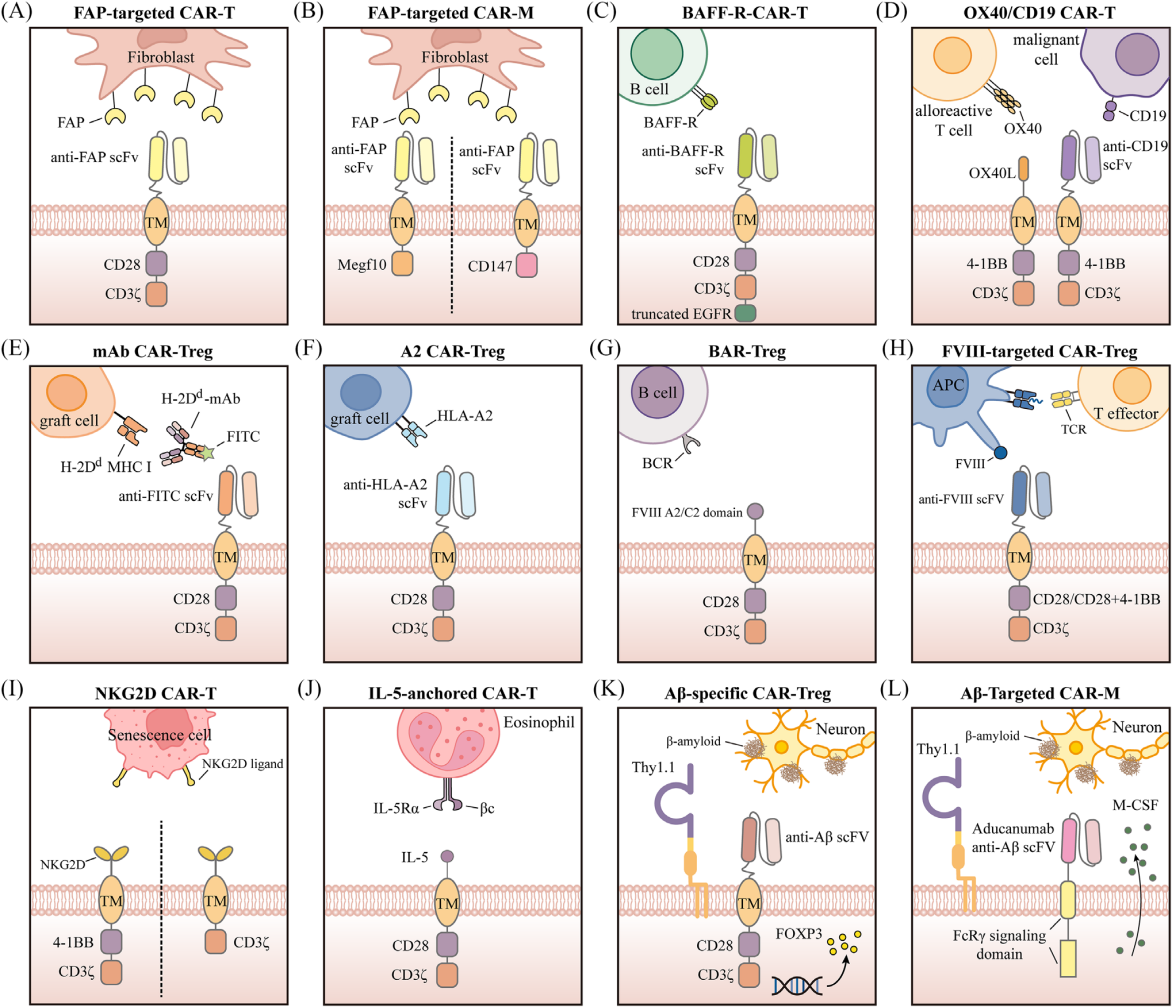
Schematic illustration of chimeric antigen receptor (CAR) constructs designed for non‐oncological diseases other than infectious and autoimmune diseases. These CAR‐engineered cells are designed with different extracellular recognition domains, enabling them to specifically target certain cells. This design facilitates the promotion of immune tolerance or the elimination of pathogenic cells while minimising damage to healthy tissues.

Cardiovascular diseases remain the leading cause of death worldwide, presenting major health risks and substantial economic burdens.^210^ Fibrosis is a common feature observed in almost every form of myocardial disease, with excessive cardiac fibrosis serving as a key contributor to the progression of both heart disease and heart failure.^211^, ^212^ However, the scarcity of clinical interventions and treatments specifically targeting fibrosis highlights the urgent need for novel therapeutic approaches.^213^ Aghajanian et al. performed gene expression analysis on human cardiac tissue from both healthy and diseased individuals, identifying fibroblast activation protein (FAP) as a potential endogenous target.^51^ Further experiments revealed that FAP is expressed by activated cardiac fibroblasts. Previous studies have shown that activated cardiac fibroblasts secrete excessive extracellular matrix, leading to fibrosis,^214^ which ultimately contributes to myocardial stiffening and impaired cardiomyocyte function.^215^ Based on these findings, the researchers investigated the use of FAP‐targeted CAR‐T cells (Figure 5A) to eliminate activated fibroblasts. The results demonstrated that FAP‐targeted CAR‐T cells significantly reduced cardiac fibrosis and improved post‐injury functional recovery in mice.^51^ To mitigate the impact of sustained anti‐fibrotic activity driven by CAR‐T on wound healing, Rurik et al. utilised lipid nanoparticles (LNPs) to deliver mRNA, generating transient, effective anti‐FAP CAR‐T cells, which notably improved heart function in a mouse model of heart failure.^216^ Interestingly, given the crucial role of stromal fibroblasts in tumour progression and metastasis, the tumour‐fighting capabilities of FAP‐targeted CAR‐T cells have been explored in previous studies.^217^, ^218^ These results indicate that anti‐FAP CAR‐T therapy could be highly effective for both oncologic and non‐oncologic diseases, though further evaluation is required to assess its efficacy and safety in humans.

CAR‐M cells possess superior tissue infiltration capabilities relative to CAR‐T cells, enabling them to more effectively penetrate dense tissues.^52^ Additionally, CAR‐M cells present a diminished risk of CRS and greater feasibility for allogeneic applications, supporting their potential role in cardiac fibrosis treatment.^219^ Recent studies have revealed that FAP‐targeted CAR‐M cells exhibit anti‐fibrotic effects similar to those of CAR‐T cells, effectively alleviating cardiac fibrosis. Gao et al. designed an anti‐FAP CAR‐M by incorporating an anti‐FAP scFv with the intracellular domain of Megf10, enabling targeted recognition of FAP and promoting phagocytic activity^52^ (Figure 5B). Their findings demonstrated that the anti‐FAP CAR effectively mediated macrophage phagocytosis of FAP‐positive target cells. Moreover, following adoptive transfer, CAR‐M cells alleviated cardiac fibrosis and improved cardiac function in mice. In another study, Wang et al. designed an anti‐FAP CAR‐M by incorporating CD147, which induces the production of matrix metalloproteinases, as the intracellular signalling domain^219^ (Figure 5B). They performed adoptive transfer of anti‐FAP CAR‐M cells into mice 3 days after ischaemia–reperfusion injury. These CAR‐M cells infiltrated the myocardium as early as 1 day post‐treatment and persisted for nearly 2 weeks. More importantly, the anti‐FAP CAR‐M cells significantly improved cardiac function in the mice, with no associated toxicity detected.

## 5 IMMUNE‐MEDIATED DISORDERS IN TRANSPLANTATION

Transplantation is the only solution or treatment option for certain end‐stage organ failures.^220^ However, patients who are prone to rejection (sensitised patients) have high levels of antibodies, which increase the likelihood of their immune systems mounting a strong rejection response against potential transplants. Consequently, these patients often have to wait longer for organ transplants.^221^ Unfortunately, due to the prolonged waiting times, such patients may die from organ failure before receiving a transplant.^222^ Research has attempted to reduce alloantibody levels in these patients through methods such as plasma exchange, monoclonal antibodies targeting B cells and the complement system, and IgG‐degrading enzymes.^223^ Nevertheless, these treatments have not shown lasting clinical benefits.^224^ A recent study suggests that CAR‐T therapy has the potential to inhibit alloantibody production, which could significantly improve transplant outcomes for sensitised patients. In this study, researchers designed CAR‐T cells capable of recognising and targeting the B‐cell activating factor receptor (BAFF‐R; Figure 5C). These CAR‐T cells precisely identify and neutralise B cells carrying BAFF‐R‐specific antigens in vitro, thereby specifically targeting and eliminating sensitised B cells.^225^

In addition to improving outcomes in solid organ transplantation, CAR‐T therapy can also benefit haematopoietic stem cell transplantation. Acute GVHD is a fatal side effect of haematopoietic stem cell transplantation,^226^, ^227^ in which OX40 is crucial in driving the expansion of host‐reactive T cells.^228^ Findings from Mo et al. indicated that bifunctional human CAR‐T cells targeting both OX40 and CD19 (Figure 5D) can protect animals from fatal xenogeneic GVHD and leukaemia relapse while preserving antiviral immunity.^229^

Notably, the successful suppression of antigen‐specific immune responses by CAR‐Tregs in various diseases provides a theoretical foundation for their application in transplant immunotolerance. Current in vitro and in vivo research has preliminarily validated the feasibility of CAR‐Treg therapy in inhibiting post‐transplant immune rejection. For instance, Pierini et al. developed a monoclonal antibody‐directed CAR (mAbCAR) system (Figure 5E) that enables Treg cells to target specific tissues through FITC labelling.^230^ Their findings demonstrated that mAbCAR Tregs targeting MHC class I proteins on allogeneic grafts not only prolonged the survival of islet grafts but also that of secondary skin grafts matching the specificity of the original islet allograft. Additionally, Dawson et al. engineered CAR‐Treg cells capable of targeting HLA‐A2+ cells^231^ (Figure 5F). These humanised A2‐CAR‐Treg cells exhibited the ability to inhibit xenogeneic GVHD driven by HLA‐A2‐positive cells and lessen the rejection response to human HLA‐A2‐positive skin grafts in vivo.

## 6 HAEMOPHILIA A

Haemophilia A is an X‐linked recessive disorder caused by a deficiency or absence of coagulation factor VIII.^232^ Currently, treatment for haemophilia A primarily involves recombinant or plasma‐derived factor VIII replacement therapy. However, due to the lack of immune tolerance to factor VIII, up to 30% of patients may develop neutralising antibodies against factor VIII during treatment.^233^, ^234^ To overcome this challenge, Parvathaneni et al. engineered both human and murine cytotoxic T cells expressing a CAR that incorporates the immunodominant FVIII domains (A2 and C2). These FVIII domain‐engineered T cells had the ability to kill factor VIII‐reactive B‐cell hybridomas, significantly reducing the production of anti‐factor VIII antibodies in haemophilic mice.^235^ These findings indicate that CAR‐T therapy holds potential as a prophylactic approach for severe haemophilia A patients at high risk of developing anti‐factor VIII antibodies. Furthermore, given that Tregs are adept at immune suppression and vital for sustaining immune tolerance and balance,^236^ extensive research has explored the use of CAR‐Treg cells to inhibit T‐ and B‐cell responses to factor VIII, achieving promising results.^237^, ^238^, ^239^, ^240^ The design of CAR‐Treg cells targeting factor VIII follows two main strategies. The first approach, known as B‐cell antibody receptor (BAR) Treg cells (Figure 5G), leverages immunodominant regions of FVIII, such as A2 and C2, enabling BAR‐Treg cells to recognise and suppress FVIII‐specific B cells. The second strategy resembles the conventional CAR‐T cell design, incorporating FVIII‐specific scFv to allow CAR‐Treg cells to recognise and target FVIII (Figure 5H). Antigen‐presenting cells carrying FVIII can facilitate the proximity of CAR‐Treg cells to effector T cells, thereby suppressing the activation of these effector T cells.

## 7 SENESCENCE‐ASSOCIATED DISEASES

Cellular senescence represents a stable and terminal state of cell cycle arrest, associated with multiple macromolecular changes and a hypersecretory, pro‐inflammatory phenotype.^241^ While senescence can act as a tumour‐suppressive mechanism by preventing the proliferation of precancerous cells^242^, ^243^ and contribute positively to wound healing,^244^, ^245^ the abnormal accumulation of senescent cells may result in the secretion of numerous inflammatory cytokines. This disrupts the structure and function of the microenvironment surrounding senescent cells, leading to chronic damage and contributing to the development of diseases such as liver and lung fibrosis, atherosclerosis, diabetes and osteoarthritis.^246^, ^247^ Studies have indicated that the clearance of senescent cells from damaged tissues in mice can alleviate tissue dysfunction and even extend lifespan.^248^, ^249^, ^250^ Currently, several small molecules capable of selectively clearing senescent cells have been identified, but most exhibit limited efficacy and significant side effects.^246^, ^251^, ^252^ CAR‐T cells, which can specifically recognise and destroy targeted cells, appear to be an ideal approach for eradicating senescent cells in the body. In a study by Amor et al., the urokinase‐type plasminogen activator receptor (uPAR) was identified as a molecule commonly upregulated on the cell surface during senescence. They then engineered CAR‐T cells specific to uPAR and discovered that these cells were capable of effectively targeting and killing senescent cells, thereby improving the senescence phenotype in mice.^253^ Aside from uPAR, natural killer group 2 member D ligands (NKG2DLs) appear to be another potential target, as they have been found to be upregulated in senescent cells as early as 2016.^254^ Two recent studies demonstrated that CAR‐T cells targeting NKG2DLs via NKG2D can effectively destroy senescent cells both in vitro and in vivo^255^, ^256^ (Figure 5I). These studies offer compelling evidence in favour of using CAR‐T therapy to treat senescence‐related diseases. Notably, the potential of glycoprotein non‐metastatic melanoma protein B (GPNMB) as a target for senescent cells has also been reported.^257^ However, further experimental evidence is needed to confirm the efficacy and safety of using GPNMB‐targeted CAR‐T cells for senescent cell elimination and senescence phenotype improvement.

## 8 ASTHMA

Asthma is a prevalent respiratory disease affecting both children and adults worldwide,^258^ with its immunopathology involving the activation of both the innate and adaptive immune systems, which drive chronic airway inflammation.^259^ Severe eosinophilic asthma (SEA) is a predominant form of refractory asthma characterised by eosinophilic inflammation, which is poorly responsive to clinical treatment.^260^ Eosinophils are key contributors to the pathogenesis of SEA, leading to airway epithelial damage and bronchial remodelling.^261^ To address this, Chen et al. designed a cytokine‐anchored CAR T cell system (Figure 5J) aimed at triggering T cell‐mediated eosinophil killing, which limited eosinophil differentiation in an asthma mouse model and provided significant protection against allergic airway inflammation.^262^ Similarly, Jin et al. designed CAR‐T cells directed against IL‐5Rα to eliminate eosinophils, ultimately achieving sustained suppression of lung inflammation and alleviation of asthma symptoms in a mouse model.^263^

## 9 NEUROLOGICAL DISORDERS

As a leading cause of disability and mortality, neurological disorders have emerged as one of the most pressing challenges in global health, urgently requiring resolution.^264^ Alzheimer's disease (AD) holds a significant position among neurological disorders, with its progressive memory loss and cognitive impairment posing a serious threat to patients’ quality of life and placing substantial strain on societal healthcare resources.^265^ Over the past few decades, numerous therapeutic strategies have been investigated for AD, yet a cure remains elusive, making prevention the primary focus.^265^, ^266^ Although the exact pathophysiological mechanisms of AD remain unclear, clinical trials involving antibodies targeting aggregated β‐amyloid (Aβ) have demonstrated that targeting Aβ can effectively reduce amyloid plaques and improve cognitive function in AD patients.^267^, ^268^ In 2016, a study by Dansokho et al., using an animal model, demonstrated that Tregs can modulate microglial responses to Aβ deposition and slow the progression of AD.^269^ This highlights the therapeutic potential of Treg cells in the context of AD. Recently, Saetzler et al. engineered Aβ‐specific CAR‐Tregs (Figure 5K), which exhibit typical Treg characteristics and show the ability to be activated in an antigen‐specific manner while suppressing the activation of CD8+ T cells in vitro.^270^ Similarly, another research team endowed Treg cells with specificity in response by designing an Aβ‐specific T‐cell receptor.^271^ Adoptive transfer of TCRAβ‐Tregs resulted in sustained immune suppression, reduced microglial activation and amyloid burden, and ultimately improved learning and memory in mice. It is worth noting that although there is a lack of specific evidence for the use of CAR‐Tregs in the treatment of Parkinson's disease, studies in animal models have shown that Tregs have a positive impact on the progression of Parkinson's disease.^272^, ^273^

A study conducted in 2022 demonstrated that peripheral blood‐derived monocytes/macrophages can infiltrate brain parenchyma and target amyloid plaques, thereby reducing plaque burden.^274^ Building on this finding, researchers have sought to utilise CAR technology to equip macrophages with the ability to specifically recognise amyloid plaques, further enhancing their clearance efficacy. Kim et al. designed CAR‐M cells capable of targeting Aβ and expressing Thy1.1 while secreting M‐CSF (colony stimulating factor) (Figure 5L), based on the monoclonal antibody aducanumab used for AD treatment.^275^ These CAR‐M cells significantly reduced amyloid plaque burden in the APP/PS1 transgenic AD mouse model.

## 10 DISCUSSION

Since its inception, CAR‐T cell treatments have shown considerable effectiveness in combating haematologic malignancies.^276^ This tremendous success in malignant tumours has sparked interest in extending the application of CAR‐T cells to diseases beyond cancer. Recent research indicates that CAR‐T cells also show great potential in treating various non‐cancerous diseases, including infectious diseases, autoimmune diseases, cardiovascular diseases and others. This discovery is undoubtedly exciting, as it means more patients can benefit from this innovative therapy. In fact, because CAR‐T therapy can specifically target and eliminate cells expressing certain antigens, it theoretically allows for the treatment of diseases by eradicating disease‐associated cell subsets. Despite the therapeutic potential of CAR‐T cell therapy for non‐oncological diseases, there remain numerous issues that require attention and further investigation.

### 10.1 Efficacy and safety

Similar to its application in cancer treatment, the durability and effectiveness of CAR‐T treatment for non‐oncological diseases remain significant challenges. Data show that 40%–60% of patients with haematological malignancies experience disease relapse after CAR‐T therapy,^277^ potentially due to the gradual exhaustion of these engineered cells in vivo, which weakens their antitumour activity.^278^ Likewise, such exhaustion may compromise the effectiveness of CAR‐T treatment for non‐oncological diseases, raising the risk of disease relapse or progression. Furthermore, since CAR‐T therapy typically targets specific antigens, it may face challenges in addressing disease heterogeneity, potentially leading to incomplete clearance of pathogenic cell populations and making disease remission difficult or prone to relapse.^278^, ^279^ To overcome these challenges, researchers have proposed various innovative strategies to improve the persistence and effectiveness of CAR‐T cells.^280^, ^281^, ^282^ However, the clinical feasibility and long‐term safety of these approaches require further in‐depth investigation and validation.

One major concern regarding CAR‐T therapy is its safety. Patients undergoing CAR‐T therapy still face some risks, such as CRS,^283^ neurotoxicity,^284^ and increased susceptibility to infections.^285^ Existing data suggest that severe toxicities are rare in autoimmune disease patients treated with CAR‐T cells, possibly due to their lower B‐cell burden compared to those with B‐cell malignancies.^149^ However, the limited data are insufficient to fully confirm the safety of CAR‐T therapy, making the improvement of its safety a primary focus. Controlling toxicity by enabling transient CAR expression through mRNA gene transfer appears to be an effective strategy.^197^ Another promising approach is the localised delivery of CAR‐T cells to concentrate them at the target site and limit their cytotoxic range, thus enhancing therapeutic outcomes while minimising potential toxicity.^286^ Furthermore, the therapeutic index of CAR‐T cells continues to be an important research priority, as precise dose control is vital to addressing safety concerns.^287^

### 10.2 Target selection

Target selection is a key factor influencing both the effectiveness and safety of CAR‐T therapy. Currently, CD19 is predominantly used as a single target in CAR‐T therapy for most autoimmune disease patients. CD19 is highly specific to the B‐cell lineage and widely present throughout various stages of B‐cell differentiation.^132^ In contrast, although CD20 has shown potential in immunotherapy for autoimmune diseases (e.g., rituximab), it is nearly absent in plasmablasts and plasma cells.^288^ BCMA and CD38 are two additional potential targets for autoimmune diseases. BCMA is mainly found on plasma cells and a subset of memory B cells, positioning it as a candidate target for antibody‐driven autoimmune diseases.^195^, ^197^, ^289^ CD38, on the other hand, is expressed on multiple immune cell populations, such as T cells, NK cells, plasma cells and early B cells.^290^ Although monoclonal antibodies targeting CD38 have shown benefits in SLE, its safety as a CAR‐T target requires further evaluation.^291^ In addition, multi‐target strategies are being actively explored. For example, dual‐target CAR‐T cells directed against both CD19 and BCMA have been developed and preliminarily validated for efficacy in SLE patients, though further research is required to evaluate their long‐term efficacy and safety.^144^ In non‐oncological diseases other than autoimmune diseases, current research primarily focuses on targeting single disease‐related antigens. The single‐target strategy offers advantages such as relatively simple design and mature technology, but it carries the risk of antigen escape. In contrast, multi‐target CAR‐T therapy may have potential advantages in enhancing efficacy and reducing the risk of relapse; however, its manufacturing process is more complex, and its long‐term safety and efficacy still require further validation.

### 10.3 Lymphodepletion

Before using CAR‐T therapy for cancer, lymphodepletion is generally considered necessary to enhance CAR‐T cell efficacy^292^, ^293^, ^294^ through mechanisms such as the removal of Treg cells, increased availability of serum cytokines and elimination of bone marrow‐derived suppressor cells.^295^, ^296^, ^297^ Pre‐treatment lymphodepletion may provide temporary benefits in CAR‐T therapy for autoimmune conditions by reducing immune cell counts, including B cells.^132^, ^141^ Currently, the most widely used lymphodepletion regimen in B‐cell malignancies is a combination of cyclophosphamide (.75–1.5 g/m^2^) and fludarabine (75–90 mg/m^2^).^298^ To date, all patients with autoimmune diseases who have undergone CAR‐T therapy have received lymphodepletion based on fludarabine and/or cyclophosphamide.^149^ However, the optimal lymphodepletion strategy for autoimmune diseases remains to be defined. A previous study suggested that lower doses of cyclophosphamide and fludarabine did not significantly impair CAR‐T cell efficacy in SLE,^299^ indicating the potential for dose reduction. This could help mitigate treatment‐related toxicity while maintaining therapeutic efficacy. Furthermore, optimising lymphodepletion regimens involves not only dose adjustments but also the selection of alternative agents. For example, bendamustine has been proposed for lymphodepletion prior to CAR‐T therapy to reduce inflammatory responses and lower toxicity risks.^300^ Future studies should further investigate how different lymphodepletion regimens influence both the initial and prolonged effectiveness of CAR‐T therapy to determine the optimal strategy.

### 10.4 Costs

A significant challenge associated with CAR‐T therapy is its high cost. These costs primarily stem from the preparation of CAR‐T cells, outpatient or inpatient care and potential treatments for adverse events.^301^ Clearly, the high cost of CAR‐T therapy limits its accessibility in low‐income regions. Moreover, many low‐income and underdeveloped areas face shortages of medical resources, including trained personnel and infrastructure, further constraining the application of CAR‐T therapy in these regions. Automating and streamlining the autologous CAR‐T cell production process, as well as reducing the time required for manufacturing, is one approach to lowering its costs. Another potential avenue is the in vivo targeting and reprogramming of T cells to express CARs, eliminating the need for ex vivo manufacturing. The in vivo editing approach not only removes the reliance on specialised facilities and personnel required for ex vivo manufacturing but also reduces the demand for cold‐chain systems and resources during transportation and storage. Beyond these two strategies, universal CAR‐T therapy seems to be another promising solution for reducing costs and improving accessibility in low‐income regions. Universal CAR‐T therapy involves the batch production of T cells derived from healthy donors, enabling long‐term storage and rapid distribution. This approach also minimises the dependence on specialised facilities and skilled personnel, making it particularly suitable for resource‐limited settings. Notably, CAR‐T therapy, if capable of providing long‐term or permanent remission, may be cost‐effective for chronic disease management compared to the cumulative expenses of long‐term drug therapy.

### 10.5 Patient selection and treatment criteria

The patient criteria, treatment prerequisites and timing of CAR‐T therapy may vary across different disease conditions. For instance, in some viral infections, such as HBV infection in the liver, the widespread distribution of the virus in specific organs may lead to large‐scale killing of infected cells by CAR‐T cells, potentially causing acute or irreversible organ damage.^302^ In the treatment of certain diseases, such as cardiac diseases, a comprehensive assessment may be required before administering CAR‐T therapy to determine whether the disease has reached an irreversible stage; otherwise, the treatment may no longer be beneficial and could even become counterproductive.^287^ Moreover, it is crucial to assess how much benefit patients can derive from CAR‐T cell therapy compared to existing treatment options. The conditions, timing and position of CAR‐T cell therapy in managing different diseases still require further clarification through experimental and clinical research.

### 10.6 Ethics considerations

CAR‐T therapy presents a range of ethical challenges, including treatment fairness, patient informed consent, ethical concerns regarding donor cells and challenges in clinical trials.^303^ Due to the high dependency on specialised equipment and personnel, the production and transportation of CAR‐T products remain limited, meaning that patients often have to wait a long time or may even miss the optimal window for treatment.^304^, ^305^ Although there has been some discussion on prioritising patients,^304^, ^305^, ^306^ determining how to more reasonably ensure fairness in treatment remains a key ethical challenge in CAR‐T therapy. Additionally, the potential adverse effects and uncertainty of treatment outcomes also pose significant risks for patients. One important ethical challenge is whether clinical researchers and physicians adequately inform research participants and patients about these risks and ensure that they make decisions based on a full understanding. Universal CAR‐T therapy, which utilises T cells from healthy donors, raises further ethical questions regarding donor consent and awareness. It is essential to ensure that donors are fully informed about the intended use of their cells and the possibility of their involvement in commercial applications. Furthermore, the risk of genetic information from donor cells being leaked during research or commercial use introduces significant privacy and data security concerns. To address these ethical challenges, a multifaceted approach is required, including robust policy interventions, stricter regulation of the informed consent process and the establishment of comprehensive mechanisms to safeguard donor privacy and manage data security effectively.

### 10.7 Combination strategies

Significant advancements in cancer treatment have been achieved by integrating CAR‐T cell therapy with other therapeutic strategies. These combination approaches aim to boost the therapeutic performance of CAR‐T cells while mitigating associated toxicity, thus broadening the therapeutic scope and improving safety. For example, combining CAR‐T therapy with immune checkpoint inhibitors has been found to improve the effectiveness of CAR‐T treatments to some degree while maintaining manageable toxicity levels.^307^, ^308^ Furthermore, the combination of CAR‐T therapy with monoclonal antibodies targeting cytokines involved in CRS or ICANS has also shown potential in mitigating CAR‐T cell toxicity.^309^ Although combination strategies involving CAR‐T therapy and other treatments for non‐oncological diseases are still in their infancy, some studies provide valuable insights that encourage further exploration of CAR‐T‐based combination therapies. One such study indicated that combining CAR‐T cells with Myrcludex B can achieve long‐term control of HBV infection in both cell and animal models.^310^ Another study has shown that combining CD19‐targeted CAR‐T therapy with Nintedanib and Mycophenolate can lead to sustained improvements in progressive pulmonary fibrosis in patients with SSc.^311^ The potential benefits of combining CAR‐T therapy with other drugs (such as immunomodulators, anti‐fibrotic agents and anti‐inflammatory drugs) to enhance treatment outcomes in various disease conditions still require further investigation. Notably, to improve the safety of CAR‐T treatment in non‐cancer diseases, lessons from cancer‐related studies can be applied, such as combining CAR‐T therapy with monoclonal antibodies or small molecules targeting cytokines associated with CRS or ICANS to manage related toxicity.

### 10.8 The potential of different CAR therapies

For non‐oncological diseases, different types of CAR‐engineered cells each have their own advantages and limitations. CAR‐T cells have demonstrated potent antigen‐specific clearance capabilities in various non‐oncological diseases, but their application is constrained by high production costs,^301^, ^312^ long manufacturing times,^313^ and severe adverse reactions.^314^ Although the development of universal CAR‐T cells presents a possible way to overcome certain challenges, their widespread use still requires addressing issues related to allogeneic cell transplantation, such as GVHD and host immune rejection.^315^, ^316^ In contrast, CAR‐NK cells, as cytotoxic lymphocytes, exhibit unique advantages in infectious diseases and autoimmune diseases due to their innate antiviral activity and ability to eliminate target cells. Additionally, CAR‐NK cells are associated with lower toxicity and possess “off‐the‐shelf” potential, as they can be derived from umbilical cord blood or cell lines.^31^, ^150^, ^151^, ^152^ However, the relatively short lifespan of NK cells, while enhancing the safety of CAR‐NK therapy, may necessitate repeated administrations to achieve long‐lasting responses.^317^, ^318^ While the role of macrophages in host defence and their exceptional tissue infiltration capabilities^52^, ^118^ make CAR‐M cells a promising candidate for infectious and cardiac diseases, their persistence and stability require further investigation. CAR‐Treg cells, as an emerging strategy, can suppress overactivated immune responses by enhancing the function of antigen‐specific Tregs,^40^ offering a novel approach to inducing immune tolerance, particularly in organ transplant rejection and autoimmune diseases. Nevertheless, it remains uncertain whether CAR‐Treg cells might induce adverse reactions similar to those of CAR‐T cells, and further evaluation is needed to determine whether the inclusion of the CD28 co‐stimulatory domain could lead to CAR‐Treg exhaustion.^313^ In brief, these CAR therapies, derived from different cell types, exhibit diverse applications due to their distinct immunological properties, potentially offering more precise and effective treatment strategies for patients.

### 10.9 Future research directions

To fully harness the potential of CAR therapies across various disease contexts, future research should focus on addressing current limitations and exploring innovative strategies for improvement. One key area is enhancing the safety of CAR therapies in both temporal and spatial dimensions through advanced CAR designs, such as various molecular switches, logic gates and transient CAR expression systems.^319^, ^320^ These approaches could minimise long‐term risks like off‐target effects and CRS while maintaining therapeutic efficacy. Another critical field requiring further exploration is the development of universal CAR‐engineered therapies. By employing gene‐editing technologies to modify one or more genes in allogeneic T cells, it is possible to effectively eliminate GVHD and immunogenicity, enabling these universal CAR‐T cells to serve as “off‐the‐shelf” products that can be broadly applied to diverse patients without the need for time‐consuming, individualised manufacturing processes.^321^ Furthermore, combining CAR therapies with other treatment modalities in non‐cancer diseases remains a promising yet underexplored area. Such combination approaches have the potential to overcome the limitations and resistance associated with monotherapies but also introduce additional complexities. Future research should focus on uncovering the synergistic mechanisms between CAR‐engineered therapies and other treatments, as well as identifying optimal combination strategies to enable their broader application in non‐cancer contexts.

**FIGURE 6:**
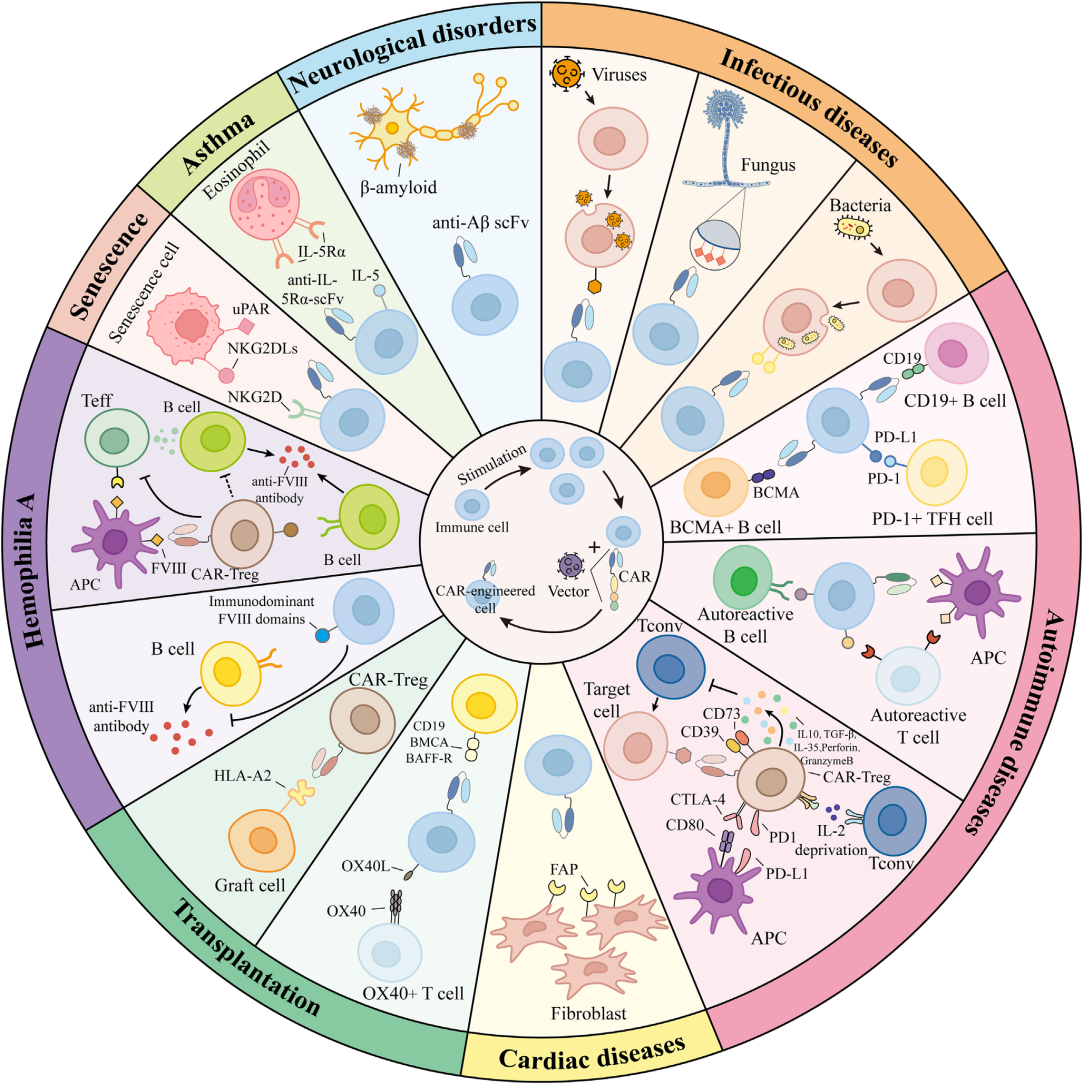
Overview of chimeric antigen receptor (CAR)‐engineered cell therapy applications in various non‐cancerous disorders. The efficacy of CAR therapy has been preliminarily explored and validated in various non‐oncological diseases, including infectious diseases, autoimmune diseases, cardiac diseases, immune‐mediated disorders in transplantation, haemophilia A, senescence‐associated diseases, asthma and neurological disorders.

To summarise, CAR‐engineered cell therapy has not only achieved significant breakthroughs in cancer treatment but is also gradually emerging as a novel approach for addressing complex non‐tumour diseases (Figure 6). With ongoing research, we can anticipate that CAR‐engineered cell therapy will continue to expand its applications, offering more options for the clinical treatment of various diseases. Looking ahead, through continuous optimisation and innovation, CAR‐engineered cell therapy is expected to overcome current technical and clinical challenges, achieve higher efficacy and safety, and ultimately benefit a broader range of patients.

## AUTHOR CONTRIBUTIONS

Lvying Wu drafted the manuscript and visualised the tables and figures. Lingfeng Zhu revised the manuscript. Jin Chen designed the review and critically revised the manuscript. All the authors read and approved the final manuscript.

## CONFLICT OF INTEREST STATEMENT

The authors declare no conflicts of interest.

## ETHICS STATEMENT

Not applicable.

## CONSENT FOR PUBLICATION

Not applicable.

## Data Availability

Data sharing is not applicable to this article as no new data were created or analysed in this study.
